# Flagellin from *Salmonella enteritidis* Enhances the Immune Response of Fused F18 from Enterotoxigenic *Escherichia coli*

**DOI:** 10.21315/tlsr2022.33.3.2

**Published:** 2022-09-30

**Authors:** An-Phuc Tran-Mai, Hong-Diep Thi Tran, Quoc-Gia Mai, Kien-Quang Huynh, Thuoc Linh Tran, Hieu Tran-Van

**Affiliations:** 1 Department of Molecular and Environmental Biotechnology, University of Science, 227 Nguyen Van Cu Street, District 5, Ho Chi Minh City, Vietnam; 2 Laboratory of Biosensors, University of Science, 227 Nguyen Van Cu Street, District 5, Ho Chi Minh City, Vietnam; 3 Vietnam National University, Vo Truong Toan Street, Linh Trung Ward, Thu Duc City, Ho Chi Minh City, Vietnam; 4 National Veterinary Joint Stock Company, 28 VSIP, Street no. 06, Vietnam-Singapore Industrial Park, Thuan An City, Binh Duong Province, Vietnam

**Keywords:** Enterotoxigenic *Escherichia coli* (ETEC), Post-Weaning Diarrhea (PWD), Flagellin Protein (FliC), F18, FliC-F18

## Abstract

F18 plays an important role in helping Enterotoxigenic *Escherichia coli* (ETEC) binds to specific receptors on small intestinal enterocytes, followed by secreting of toxins causing diarrhea in post-weaning piglets (post-weaning diarrhea, PWD). However, the F18 subunit vaccine is not sufficient to stimulate an immune response that can protect weaning pigs from F18-positive ETEC (F18^+^ETEC). Recently, a body of evidence shows that flagellin protein (FliC) helps to increase the immunity of fused proteins. Therefore, in this study, we combined FliC with F18 to enhance the immune response of F18. The *f18* gene was obtained from F18^+^ETEC, then was fused with the *fliC* gene. The expression of recombinant FliC-F18 protein was induced by Isopropyl-beta-D-Thiogalactopyranoside (IPTG). The purified protein was tested *in vivo* in mouse models to evaluate the immunostimulation. Results showed that the fusion of FliC and F18 protein increased the production of anti-F18 antibodies. Besides, the anti-F18 antibody in the collected antiserum specifically identified F18^+^ETEC. This result provides proof-of-concept for the development of subunit vaccine to prevent PWD using F18 antigen.

**Highlights**
- FliC-F18 was recombinantly expressed.
- FliC-F18 increased the production of anti-F18 antibodies without complete adjuvant.
- The isolated antibodies specifically identified F18^+^ETEC.

## INTRODUCTION

Post-weaning diarrhea (PWD) is one of the most common threats to the swine industry worldwide (Rhouma *et al*. 2017). The cause of PWD is associated with the proliferation of ETEC in the pig intestine. Most *Escherichia coli* strains involved PWD express F4 (K88) or F18 fimbriae (Luise *et al*. 2019; Nagy & Fekete 1999), which are important virulence factors. They allow ETEC to bind to specific receptors on small intestinal enterocytes, followed by colonisation and secretion of enterotoxins causing diarrhea. Therefore, one of PWD prevention strategies is using vaccines against F4^+^ETEC and F8^+^ETEC. Although vaccines against F4 provide good protection from the PWD caused by F4^+^ETEC, vaccines against F18 have not shown promising results due to poor immune response and difficulty producing specific antibodies (Delisle *et al*. 2012; Melkebeek *et al*. 2013; Verdonck *et al*. 2007). Hence, there is an unmet need for research on increasing the immune response of F18 as well as providing complete protection from PWD. In different researches, the immunogenicity of FliC protein was reported. In particular, FliC can stimulate a humoral immune response against the fused protein (Tran *et al*. 2020). With that in mind, we speculated that if FliC were an immuno-modulator, it would enhance the immune response against F18 without a complete adjuvant. Therefore, in this study, F18 had been fused with the FliC protein to enhance the immune response of F18. In particular, FliC was an expected protein-based adjuvant to enhance the immune response of F18 in the host body. The results of this study pave the way for the development of a preventive vaccine against PWD caused by ETEC, especially antigens with low immunogenic properties like F18.

## MATERIALS AND METHODS

### Bacterial Strains, Plasmids and Growth Conditions

*E. coli* DH5α [F− endA1 hsdR17 (rk−/mk−) supE44 thi λ-recA1 gyrA96 ΔlacU169 (ϕ80 lacZ ΔM15)] and *E. coli* BL21(DE3) (F+ ompT hsdSB (rB− mB−) gal dcm(DE3) were used as host strains for cloning and protein expression, respectively. Both were cultured in Luria-Bertani (LB) medium containing 30 μg/mL of ampicillin at 37°C. F4 enterotoxigenic *E. coli* (F4^+^ETEC) and F18 enterotoxigenic *E. coli* (F18^+^ETEC) were kindly provided by Dr. Eric Cox (Ghent University). F18^+^ETEC and pET-22b/*fliC* plasmid (Tran *et al*. 2020) were used to obtain the genes encoding for F18 and FliC proteins, respectively. pET-22b was used as a cloning vector for the *f18* gene to produce pET-22b/*f18* and pET22b/*fliC-f18* recombinant plasmids, and protein expression controlled by T7 promoter via the IPTG (Isopropyl beta-D-1thiogalactopyranosie) inducer (Biobasic). F4^+^ETEC was used in the cross-reaction experiment.

### Construction of pET-22b/*f18* Recombinant Plasmid

The *f18* gene was obtained from the genome of F18^+^ETEC (GenBank: GQ325633.1) (Barth *et al*. 2011) by polymerase chain reaction (PCR) with specific primers F18F and F18R (Table 1). The pET22b plasmid was digested with *Nde*I and *Xho*I (Thermo Scientific, USA) to create sticky ends, followed by a recombinase-free cloning (RFC) protocol (Vo-Nguyen *et al*. 2022). The resulting mixture was transformed into *E. coli* DH5α competent cells. The transformants were initially screened on an ampicillin containing LB agar plate (LBAmp), then screened again by colony PCR with primers F18F/F18R and T7pro/T7ter. Plasmids collected from positive colonies were checked by the structure using digestion reaction with *Nde*I and *Xho*I, before sending for sequencing.

### Construction of pET-22b/*fliC-f18* Recombinant Plasmid

The *f18* gene was obtained from the genome of F18^+^ETEC by PCR with specific primers F18fF and F18R (Table 1). The *f18* gene and pET-22b/*fliCgfp* plasmid (Tran *et al*. 2015) were digested with *Bam*HI and *Xho*I (Thermo Scientific). The digested result of the pET22b/*fliCgfp* plasmid was separated by agarose electrophoresis, followed by the collection of the gel band containing digested pET22b/*fliC*. The gel was treated immediately after collection, then was ligated with digested *f18* gene by T4 DNA Ligase (Thermo Scientific, US) to the C-terminal of FliC. The resulting mixture was transformed into competent *E. coli* DH5α. All the screening steps were the same with the construction of pET-22b/*f18* recombinant plasmid, but replace primers F18F with F18fF, and replace *Nde*I with *Bam*HI.

### Expression of FliC-F18, FliC and F18 in *E. coli* BL21(DE3)

The protein expression was conducted as described with some modifications (Ausubel 2003). pET22b/*fliC-f18*, pET22b/*fliC* and pET22b/*f18* were transformed into competent *E. coli* BL21(DE3). Colonies that grew on LBAmp agar plates were re-screened by colony PCR with specific primers (F18F/F18R and F18fF/F18R). After screening steps, positive colonies were inoculated in 5 ml LBAmp media shaking tubes and allowed to grow at 37°C in 16 h, followed by sub-cultured at 1:10 (v/v). After OD_600_ reached 0.8–1.0, the cultures were induced with 0.1 mM of IPTG. The protein expressions were performed overnight at 16°C. The expression results were analysed by SDS-PAGE and Coomassie Brilliant Blue stained, followed by Western blot and probed with anti-His-tag antibody (Santa Cruz).

### SDS-PAGE and Western Blotting

SDS-PAGE and Western blotting were performed as described with some modifications (Ausubel 2003). The protein expression was confirmed by analysing in 12.5% SDS-PAGE gel, followed by Western blotting. The proteins from the gel were transferred to the nitrocellulose membrane before probing with mouse-anti-His-tag antibody (Santa Cruz) and finally, detecting by rabbit-anti-mouse IgG-HRP (Santa Cruz).

### Purification of FliC-F18, F18 and FliC Proteins

FliC-F18, F18 and FliC proteins were purified as described with some modification (Ausubel 2003). The biomass of 100 mL *E. coli* BL21(DE3)/pET22b/*f18* (or pET22b/*fliC-f18* or pET-22b/*fliC*) was sonicated on ice to get the supernatant containing soluble proteins that were used as raw material to purify F18 (or FLiC-F18, or FliC) by affinity chromatography with HP Hitrap column (GE Healthcare, USA). After being equilibrated with a binding buffer (50 mM TrisHCl, 100 mM NaCl, 20 mM imidazole pH 8.0), the column was loaded with the soluble protein solution, then equilibrated and washed with the binding buffer. After that, an elution buffer (50 mM TrisHCl, 100 mM NaCl, 200 mM imidazole pH 8.0) was added to elute the target protein.

The purity of F18 (or FLiC-F18, or FliC) was tested by SDS-PAGE, followed by Coomassie Brilliant Blue stained and GelAnalyzer software. Finally, purified F18 (or FLiC-F18, or FliC) was dialysed, concentrated and measured by the Bradford method.

### Immunisation with FliC-F18, F18 and FliC Proteins

Animals were maintained and performed in the experimental animal facility, and experiments by the Directive 2010/63/EU guideline approved by The Animal Care and Use Committee of University of Science, Vietnam. The total of 12 healthy male white mice (*Mus musculus* var. Albino), 3–5 weeks old, 18 g–20 g on average, were divided into four groups (3 mice/group). The blood of these mice was collected two days before the first dose injection. Groups 1, 2, 3 and 4 were injected with 25 ng FliC + 50 μL FCA (Freund’s Complete Adjuvant, Santa Cruz), 25 ng F18 + 50 μL FCA, 25 ng F18 + 50 μL FIA (Freund’s Incomplete Adjuvant, Santa Cruz), and 25 ng FLiC-F18 + 50 μL FIA, respectively. Booster doses were injected after one week, then repeated every one week with half the amount of antigen, but the same amounts of adjuvants. The amount of antigen protein used for injection was calculated based on the purity of that protein. Five days after each injection, 200 μL–300 μL of blood was obtained from the tail vein of each mouse; particularly for the last blood collection, 300 μL–400 μL of blood was collected from the orbital sinus of each mouse. The serum was collected and stored at −20°C for later analysis.

### Evaluation of the Immune-Stimulatory Effect of F18 and FLiC-F18 by Indirect ELISA (Enzyme-Linked Immunosorbent Assay)

Specific antibody titers of four groups were determined by indirect ELISA as described with some modifications (Ausubel 2003). Each well of the 96 well microtiter plate was coated with 20 μg/mL F18 in 100 μL coating buffer at room temperature in 2 h, followed by blocking with 100 mL of 5% skim milk in PBST for 1 h at room temperature. After three times of washing with PBST, 100 μL of antisera collected from the four groups, diluted in a 2-fold dilution manner from 1:200 to 1:25,600 were added and incubated at room temperature for 1 h. After removing the solution, 100 μL/well of rabbitantimouse IgGHRP at 1:5,000 dilution was added and incubated for 1 h at room temperature. 100 μL of 3, 3′, 5, 5′ tetramethyl benzidine (TMB) was added to each well and incubated for 8 min at room temperature. The total of 50 μL of H_2_SO_4_ 1 M was added to each well to stop the reaction. Finally, the absorbance at 450 nm was measured on a microplate reader. Negative control was performed in a completely similar manner but replaced with the serum collected before the first injection.

### Evaluation of the Interaction between Anti-F18 Antisera and ETECs

Dot blot was performed according to the Dot Blotter 48 Clever (CSL-D48 - Dot Blotter, 48 Samples) protocol with some modifications. The total of 0.5 μL of F18^+^ETEC was loaded on the nitrocellulose membrane of dots 1, 2 and 3, whereas *E. coli* DH5α was loaded on the nitrocellulose membrane of dots 4 and 5. Then the membranes were blocked with 5% skim milk at room temperature for 1 h. For the primary antibody, dot 1 was loaded 50 μL antisera containing anti-F18 antibodies collected from F18^+^ETEC infected pig (kindly provided by Professor Eric Cox, Ghent University); dots 2 and 4 were loaded 50 μL of 1:200 diluted antisera which were collected from FLiC-F18 + FIA injected group (group 4); dots 3 and 5 were loaded PBS buffer (1.42 g Na_2_HPO_4_; 0.24 g KH_2_PO_4_; 8 g NaCl; 0.2 g KCl; prepared in 1 L, pH 7.4). After incubating for 1 h at room temperature, the membranes were washed five times with PBST before accordingly adding antimouse IgGHRP (at 1/5,000 dilution in PBST), and goat antiswine IgGHRP (at 1/1,000 dilution in PBST). These secondary antibodies were incubated for 1 h at room temperature before washing five times with PBST. Finally, TMB substrate was added to observe the signal. Also, the crossreaction experiment was performed similarly but using F4^+^ETEC instead of F18^+^ETEC.

## RESULTS

### Molecular Cloning of Recombinant pET-22b/*f18*

**Figure 1:**
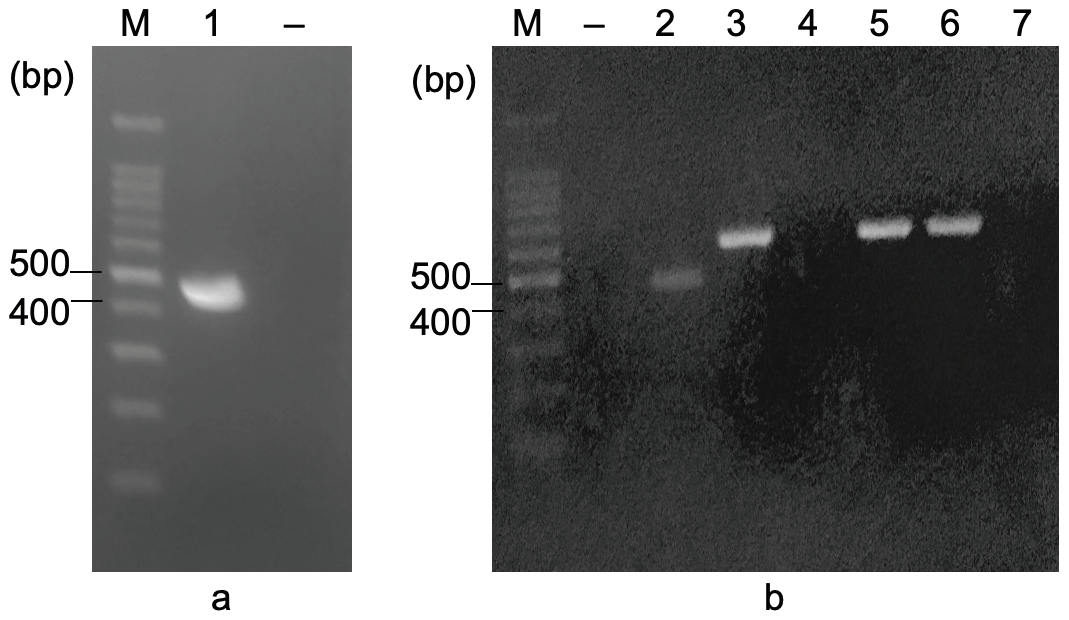
*f18* gene obtained from (a) F18^+^ETEC genome and (b) colony PCR *E. coli* DH5a/ pET22b/*f18*. M = DNA ladder; (−) = negative control; 1, 2 = *f18*; 3–7 = candidate colonies.

After PCR for collecting *f18*, there was one band between 400 bp and 500 bp on the gel (lane 1, Fig. 1a), which was the predicted size of the *f18* gene (450 bp). Whereas there was no band on the negative control (PCR reaction without F18^+^ETEC genome) (lane −, Fig. 1a), which shows that the PCR reactions were not contaminated. Therefore, the *f18* gene was successfully collected.

pET-22b plasmid after ligated with *f18* gene were transformed into *E. coli* DH5a. Five colonies grown on LBAmp medium were chosen to perform colony PCR with F18F primer (which bind to 5′ end of *f18* gene) and T7ter primer (which bind to T7 terminator) to confirm the presence of pET22b/*f18* plasmid. Three of five colonies contained pET22b/*f18* plasmid which showed as the bands approximately 582 bp (lanes 3, 5 and 6, Fig. 1b), including the *f18* gene (450 bp) incorporated with the length from T7 terminator to the recognition site of the *Xho*I enzyme on the plasmid (132 bp). In contrast, there was no amplicon band in the negative control (lane −, Fig. 1b). Therefore, the desired recombinant vector was confirmed.

### Molecular Cloning of Recombinant pET22b/*fliCf18*

**Figure 2:**
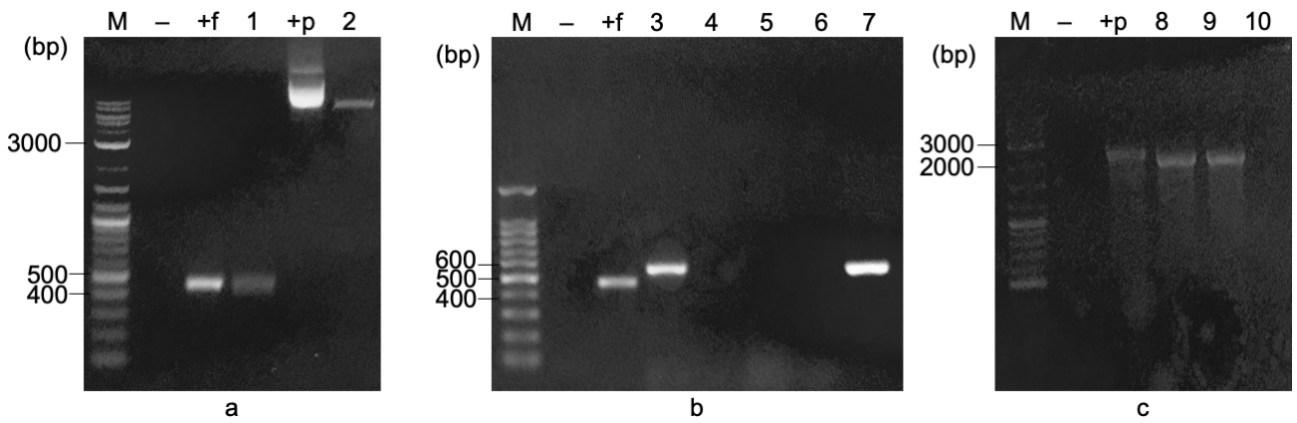
*f18* and pET22b/*fliCgfp* after digesting with (a) *Bam*HI and *Xho*I, (b) colony PCR of *E. coli* DH5α/pET22b/*fliC-f18* with 18F/T7ter and (c) T7pro/T7ter primer. M = DNA ladder; (−) = negative control; (+f) = *f18*; (+p) = pET22b/*fliCgfp;* 1 = digested *f18;* 2 = digested pET22b/*fliCgfp*; 3–10 = candidate colonies.

The *f18* gene after digested with *Bam*HI and *Xho*I had only one band of approximately 450 bp (lane 1, Fig. 2a), which was nearly the same size as a positive control containing the *f18* gene (lane +f, Fig. 2a). Also, pET22b/*fliCgfp* plasmid after digested with *Bam*HI and *Xho*I showed 2 bands at approximately 6,800 bp and 700 bp, corresponding to linear pET22b/*fliC* plasmid (6,867 bp) and *gfp* fragment (715 bp) (lane 2, Fig. 2a). pET22b/*fliC* linear plasmid was collected from the gel, then was ligated with *f18* gene by DNA T4 Ligase. The ligation result was transformed into *E. coli* DH5α. Colonies that grew on LBAmp plate were performed colony PCR with F18F primer (which bind to 5′ end of *f18*) and T7ter primer (which bind to T7 terminator of plasmid pET22b/*fliC-f18*) to confirm the presence of pET22b/*fliC-f18*. Amplicons were about 582 bp (lanes 3 and 7, Fig. 2b), including the *f18* gene (450 bp) and the fragment from the T7 terminator to the restriction site of *Xho*I on the plasmid (132 bp). When PCR with T7pro and T7ter primers, the resulting band was approximately 2,190 bp (lanes 8–9, Fig. 2c), including *fliC* gene (1,515 bp), *f18* gene (450 bp), the DNA fragment from T7 promoter of the plasmid to the starting point of *fliC* gene (100 bp), and the DNA fragment from the endpoint of *f18* gene to T7 terminator of the plasmid (125 bp). Therefore, the recombinant pET22b/*fliC-f18* was confirmed.

### Expression of FliC-F18, FliC and F18

**Figure 3:**
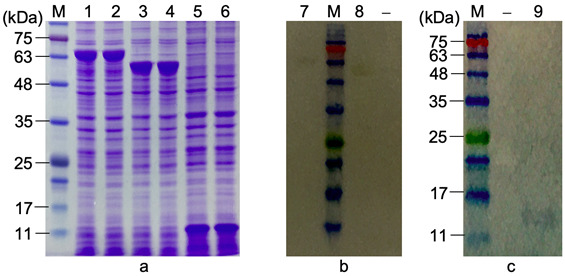
The expression of FLiC-F18, FliC and F18 was analysed by (a) SDS-PAGE and (b, c) Western blot with anti-His-tag antibody. M = protein ladder; 1, 2 = total protein and soluble fraction of *E. coli* BL21(DE3)/pET22b/*fliCf18* + IPTG; 3, 4 = total protein and soluble fraction of *E. coli* BL21(DE3)/pET22b/*fliC* + IPTG; 5, 6 = total protein and soluble fraction of *E. coli* BL21(DE3)/pET22b/*f18* + IPTG; (−) = negative control (no anti-His-tag antibody); 7 = FliC-F18; 8 = FliC; 9 = F18.

The pET22b/*fliC-f18*, pET22b/*fliC* and pET22b/*f18* plasmids were transformed into *E. coli* BL21(DE3) competent cells. Positive clones were induced by IPTG to produce target proteins. After verifying by SDS-PAGE and Coomassie brilliant blue staining (Fig. 3a), there were overexpression bands on the gel of about 67 kDa (lanes 1, 2), 52 kDa (lanes 3, 4), and 15 kDa (lanes 5, 6), which were exactly the predicted sizes of FLiC-F18, FliC, and F18, respectively. Besides, the recombinant proteins were fused with 6×His-tag, so the expression of these proteins could be indirectly confirmed by Western blot with His-tag antibody (Figs. 3b and 3c). The result showed that the over-expressed bands in the SDS-PAGE gel were FLiC-F18, FliC and F18. Thus, FLiC-F18, FliC and F18 recombinant proteins had successfully expressed in *E. coli* BL21(DE3).

### Purification of FliC-F18, FliC and F18 Proteins

**Figure 4:**
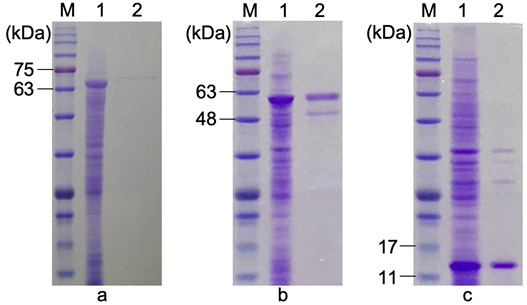
Purity evaluation of (a) FLiC-F18, (b) FliC and (c) F18 by SDS-PAGE and Coomassie brilliant blue staining. M = protein ladder; 1 = total sample of FLiC-F18, FliC, F18; 2 = purified FLiC-F18, FliC, F18.

With the fused His-tag, FLiC-F18, FliC and F18 were purified using HisTrap column. The protein purity was then evaluated by GelAnalyzer software. The results showed that the purity of FLiC-F18, FliC and F18 was 98%, 80% and 84.5%, respectively (Fig. 4). Although the yield for FLiC-F18 was quite low compared to other two, the target proteins were successfully purified and collected to use for immunological evaluation. Besides, F18 purification efficiency was still low, further investigation in purification protocol is necessary for the large amount production of this protein.

### Evaluation of the Immune-Stimulatory Effect of FliC-F18

**Figure 5:**
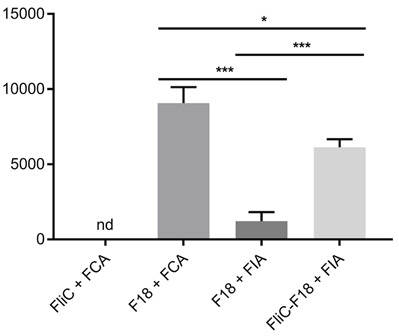
Evaluation of anti-F18 antibody titers. FCA = Freund’s Complete Adjuvant; FIA = Freund’s Incomplete Adjuvant; nd = not detected; * *p* < 0.05; *** *p* < 0.001.

The F18 specific antibody titers in collected antisera from mice groups were determined via indirect ELISA to evaluate the ability of FliC in enhancing the immune response of the F18 antigen. The OD^450 nm^ of serum samples at the beginning of this experiment were 0.065 ± 0.005, which was similar to the background signal (0.055 ± 0.005) (control sample using PBST instead of antisera). This result meant that there was no anti-F18 antibody before F18 antigen injection. In the antisera of group 1 (FliC + FCA), there was no anti-F18 antibody as well (Fig. 5). The antibody titer in group 4 (FLiC-F18 + FIA) was five times higher than in group 3 (F18 + FIA), and only 0.6 times lower than group 2 (F18 + FCA) (Fig. 5). This result indicated that the fusion of F18 with FliC had increased the anti-F18 antibody production compared to F18 + FIA.

### Evaluation of the Cross-reaction between Anti-F18 Antisera and ETECs

**Figure 6:**
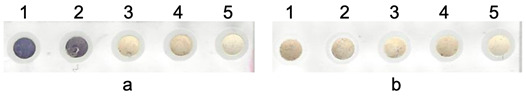
Cross-reaction of collected anti-F18 antibodies with (a) F18^+^ETEC and (b) F4^+^ETEC. 1 = F18^+^ETEC (A)/F4^+^ETEC (B) + antisera containing anti-F18 antibodies collected from F18^+^ETEC infected pig + anti-swine IgG-HRP; 2 = F18^+^ETEC (A)/F4^+^ETEC (B) + antisera containing anti-F18 antibodies collected from FliC-F18+FIA injected mouse + anti-mouse IgG-HRP; 3 = F18^+^ETEC (A)/F4^+^ETEC (B) + PBS + anti-swine IgG-HRP + anti-mouse IgG-HRP; 4 = *E. coli* DH5a + antisera containing anti-F18 antibodies collected from FliC-F18+FIA injected mouse + anti-mouse IgG-HRP; 5 = *E. coli* DH5a + PBS + anti-swine IgG-HRP + anti-mouse IgG-HRP.

This experiment evaluated the reaction of the anti-F18 antibody in antisera collected from FliC-F18+FIA injected group with F18^+^ETEC and tested the cross-reactivity of the anti-F18 antibody with F4^+^ETEC as well. In the case of F18^+^ETEC, the colourimetric signal generation was seen on dots 1 and 2 but did not appear on the others (Fig. 6a). In contrast, there was no signal generation when testing with F4^+^ETEC (Fig. 6b). This indicated that antisera collected from FLiC-F18 + FIA injected group did not have cross-reaction with F4^+^ETEC. In other words, antisera collected from the FliC-F18 + FIA injected group was specific to the F18 antigen.

## DISCUSSION

Vaccines against F18 have not shown promising results due to poor immune response and difficulty producing specific antibodies (Delisle *et al*. 2012; Melkebeek *et al*. 2013; Verdonck *et al*. 2007). We found that FliC enhanced the immune response of fused F18 protein. In detail, the antibody titers of (FliC-F18 + FIA) injected group was five times higher than (F18 + FIA) injected group. Other researchers also confirm the stimulating effect of FliC and its adjuvant activity in the context of fusion proteins. Ruth Arnon’s group pointed out that heterologous peptide sequences inserted into FliC induced humoral immunity in the absence of an adjuvant (Ben-Yedidia *et al*. 1999; Jeon *et al*. 2002; Levi & Arnon 1996; McEwen *et al*. 1992). Honko’s research remarked that FliC dramatically increased anti-F1 plasma IgG titers while the nonFliC group had undetectable antibody responses (Honko *et al*. 2006). Moreover, FliC was used in a broad range of recombinant vaccines (Adar *et al*. 2009; Bargieri, Leite, *et al*. 2011; Bargieri, Rosa, *et al*. 2008; Cuadros *et al*. 2004; das Graças Luna *et al*. 2000; Delaney *et al*. 2010; Huleatt, Jacobs, *et al*. 2007; Huleatt, Nakaar, *et al*. 2008; Mizel *et al*. 2009; Pereira *et al*. 2001; Song *et al*. 2009). The effectiveness of FliC could come from its ability to promote the TLR5 related innate-immune processes, such as induce lymphoid and non-lymphoid cells to secrete pro-inflammatory cytokine and chemokines; recruit T and B lymphocytes to secondary lymphoid sites; activate dendritic cells, TLR5^+^CD11c^+^ cells, and T lymphocytes (Mizel & Bates 2010).

Vaccination against F18^+^ETEC is an unsolved problem. All strategies include oral live and subunit vaccines as well as encapsulated vaccines, or parenteral vaccines stills had limited success until now (Felder *et al*. 2000; Verdonck *et al*. 2007). Therefore, this result could be a promising strategy to use FLiC-F18 as a parenteral vaccine for both pregnant sows and suckling pigs to protect neonates and post-weaning pigs against F18^+^ETEC infection. However, the cause of PWD is a non-invasive infection, thus mucosal immunity with F4/F18specific IgA plays an important role in preventing this disease. Further researches could focus on FliC-F18 based oral vaccines for post-weaning pigs because FliC had the potential to promote humoral immune response by oral immunisation (Salman *et al*. 2009; Synnott *et al*. 2009). In summary, our results as well as those of other researchers demonstrated that FliC-F18 is a good strategy for immunoprophylaxis of PWD caused by F18^+^ETEC.

## CONCLUSION

The immunostimulation of F18 was robustly enhanced by fusing with FliC protein. The anti-F18 antibodies in the immunised mice significantly increased and specifically recognised the F18^+^ETEC. This study is a prototype of the F18 subunit vaccine development to prevent PWD associated with F18^+^ETEC. However, further research is needed to demonstrate the complete protection of FliC-F18 in the immunised mice and piglets.

## Figures and Tables

**Table 1:** Primers used in this study.

Gene (bp)	Sense	Sequence (5′–3′)	Reference
*f18* (450)	F18F	aactttaagaaggagatata**catatg**cagcaaggggatgttaaa	This study
	F18R	cagtggtggtggtggtggtg**ctcgag**cttgtaagtaaccgcgtaagcc	This study
	F18fF	**cgcggatcc**ggtcagcaaggggatgttaaatt	This study

*Note*: bold and underlined letters indicate restriction enzyme.

## References

[b1-tlsr-33-3-19] Adar Y, Singer Y, Levi R, Tzehoval E, Perk S, Banet-Noach C, Nagar S, Arnon R, Ben-Yedidia T (2009). A universal epitope-based influenza vaccine and its efficacy against H5N1. Vaccine.

[b2-tlsr-33-3-19] Ausubel FM (2003). Current protocols in molecular biology, Suppl. 64.

[b3-tlsr-33-3-19] Bargieri DY, Leite JA, Lopes SC, Sbrogio-Almeida ME, Braga CJ, Ferreira LC, Soares IS, Costa FT, Rodrigues MM (2010). Immunogenic properties of a recombinant fusion protein containing the C-terminal 19 kDa of Plasmodium falciparum merozoite surface protein-1 and the innate immunity agonist FliC flagellin of *Salmonella typhimurium*. Vaccine.

[b4-tlsr-33-3-19] Bargieri DY, Rosa DS, Braga CJ, Carvalho BO, Costa FT, Espíndola NM, Vaz AJ, Soares IS, Ferreira LC, Rodrigues MM (2008). New malaria vaccine candidates based on the Plasmodium vivax Merozoite Surface Protein-1 and the TLR-5 agonist *Salmonella typhimurium* FliC flagellin. Vaccine.

[b5-tlsr-33-3-19] Barth S, Schwanitz A, Bauerfeind R (2011). Polymerase chain reaction-based method for the typing of F18 fimbriae and distribution of F18 fimbrial subtypes among porcine Shiga toxin-encoding *Escherichia coli* in Germany. Journal of Veterinary Diagnostic Investigation.

[b6-tlsr-33-3-19] Ben-Yedidia T, Marcus H, Reisner Y, Arnon R (1999). Intranasal administration of peptide vaccine protects human/mouse radiation chimera from influenza infection. International Immunology.

[b7-tlsr-33-3-19] Cuadros C, Lopez-Hernandez FJ, Dominguez AL, McClelland M, Lustgarten J (2004). Flagellin fusion proteins as adjuvants or vaccines induce specific immune responses. Infection and Immunity.

[b8-tlsr-33-3-19] das Graças Luna M, Sardella FF, Ferreira LC (2000). *Salmonella* flagellin fused with a linear epitope of colonization factor antigen I (CFA/I) can prime antibody responses against homologous and heterologous fimbriae of enterotoxigenic *Escherichia coli*. Research in Microbiology.

[b9-tlsr-33-3-19] Delaney KN, Phipps JP, Johnson JB, Mizel SB (2010). A recombinant flagellin-poxvirus fusion protein vaccine elicits complement-dependent protection against respiratory challenge with vaccinia virus in mice. Viral Immunology.

[b10-tlsr-33-3-19] Delisle B, Calinescu C, Mateescu MA, Fairbrother JM, Nadeau E (2012). Oral immunization with F4 fimbriae and CpG formulated with carboxymethyl starch enhances F4-specific mucosal immune response and modulates Th1 and Th2 cytokines in weaned pigs. Journal of Pharmacy and Pharmaceutical Sciences.

[b11-tlsr-33-3-19] Felder CB, Vorlaender N, Gander B, Merkle H, Bertschinger H (2000). Microencapsulated enterotoxigenic *Escherichia coli* and detached fimbriae for peroral vaccination of pigs. Vaccine.

[b12-tlsr-33-3-19] Honko AN, Sriranganathan N, Lees CJ, Mizel SB (2006). Flagellin is an effective adjuvant for immunization against lethal respiratory challenge with *Yersinia pestis*. Infection and Immunity.

[b13-tlsr-33-3-19] Huleatt JW, Jacobs AR, Tang J, Desai P, Kopp EB, Huang Y, Song L, Nakaar V, Powell T (2007). Vaccination with recombinant fusion proteins incorporating Toll-like receptor ligands induces rapid cellular and humoral immunity. Vaccine.

[b14-tlsr-33-3-19] Huleatt JW, Nakaar V, Desai P, Huang Y, Hewitt D, Jacobs A, Tang J, McDonald W, Song L, Evans RK (2008). Potent immunogenicity and efficacy of a universal influenza vaccine candidate comprising a recombinant fusion protein linking influenza M2e to the TLR5 ligand flagellin. Vaccine.

[b15-tlsr-33-3-19] Jeon SH, Ben-Yedidia T, Arnon R (2002). Intranasal immunization with synthetic recombinant vaccine containing multiple epitopes of influenza virus. Vaccine.

[b16-tlsr-33-3-19] Levi R, Arnon R (1996). Synthetic recombinant influenza vaccine induces efficient long-term immunity and cross-strain protection. Vaccine.

[b17-tlsr-33-3-19] Luise D, Lauridsen C, Bosi P, Trevisi P (2019). Methodology and application of *Escherichia coli* F4 and F18 encoding infection models in post-weaning pigs. Journal of Animal Science and Biotechnology.

[b18-tlsr-33-3-19] McEwen J, Levi R, Horwitz RJ, Arnon R (1992). Synthetic recombinant vaccine expressing influenza haemagglutinin epitope in *Salmonella* flagellin leads to partial protection in mice. Vaccine.

[b19-tlsr-33-3-19] Melkebeek V, Goddeeris BM, Cox E (2013). ETEC vaccination in pigs. Veterinary Immunology and Immunopathology.

[b20-tlsr-33-3-19] Mizel SB, Bates JT (2010). Flagellin as an adjuvant: cellular mechanisms and potential. The Journal of Immunology.

[b21-tlsr-33-3-19] Mizel SB, Graff AH, Sriranganathan N, Ervin S, Lees CJ, Lively MO, Hantgan RR, Thomas MJ, Wood J, Bell B (2009). Flagellin-F1-V fusion protein is an effective plague vaccine in mice and two species of nonhuman primates. Clinical and Vaccine Immunology.

[b22-tlsr-33-3-19] Nagy B, Fekete PZ (1999). Enterotoxigenic *Escherichia coli* (ETEC) in farm animals. Veterinary Research.

[b23-tlsr-33-3-19] Pereira CM, Guth BEC, Sbrogio-Almeida ME, Castilho BA (2001). Antibody response against *Escherichia coli* heat-stable enterotoxin expressed as fusions to flagellin. Microbiology.

[b24-tlsr-33-3-19] Rhouma M, Fairbrother JM, Beaudry F, Letellier A (2017). Post weaning diarrhea in pigs: risk factors and non-colistin-based control strategies. Acta Veterinaria Scandinavica.

[b25-tlsr-33-3-19] Salman HH, Irache JM, Gamazo C (2009). Immunoadjuvant capacity of flagellin and mannosamine-coated poly (anhydride) nanoparticles in oral vaccination. Vaccine.

[b26-tlsr-33-3-19] Song L, Zhang Y, Yun NE, Poussard AL, Smith JN, Smith JK, Borisevich V, Linde JJ, Zacks MA, Li H (2009). Superior efficacy of a recombinant flagellin: H5N1 HA globular head vaccine is determined by the placement of the globular head within flagellin. Vaccine.

[b27-tlsr-33-3-19] Synnott A, Ohshima K, Nakai Y, Tanji Y (2009). IgA response of BALB/c mice to orally administered *Salmonella typhimurium* flagellin-displaying T2 bacteriophages. Biotechnology Progress.

[b28-tlsr-33-3-19] Tran B-CT, Vo-Nguyen H-V, Nguyen V-A, Tran TL, Tran-Van H (2020). FliC-delta220-320 from *Salmonella enteritidis* as an adjuvant for vaccine development. SSR Institute of International Journal of Life Sciences.

[b29-tlsr-33-3-19] Verdonck F, Tiels P, Van Gog K, Goddeeris B, Lycke N, Clements J, Cox E (2007). Mucosal immunization of piglets with purified F18 fimbriae does not protect against F18+ *Escherichia coli* infection. Veterinary Immunology and Immunopathology.

[b30-tlsr-33-3-19] Vo-Nguyen H-V, Nguyen T-T, Mai Q-G, Tran T-T, Tran TL, Tran-Van H (2022). Recombinase-free cloning (RFC) protocol for gene swapping. Molecular Biology Research Communications.

